# Agreement of Different Drug-Drug Interaction Checkers for Proton Pump Inhibitors

**DOI:** 10.1001/jamanetworkopen.2024.19851

**Published:** 2024-07-09

**Authors:** Massimo Carollo, Salvatore Crisafulli, Margherita Selleri, Luca Piccoli, Luca L’Abbate, Gianluca Trifirò

**Affiliations:** 1 Department of Diagnostics and Public Health, University of Verona, Verona, Italy; 2 Department of Medicine, University of Verona, Verona, Italy

## Abstract

**Importance:**

Proton pump inhibitors (PPIs) are a widely prescribed class of drugs, potentially interacting with a large number of medicines, especially among older patients with multimorbidity and polypharmacy. Beyond summary of product characteristics (SPCs), interaction checkers (ICs) are routinely used tools to help clinicians in medication review interventions.

**Objective:**

To assess the consistency of information on drugs potentially interacting with PPIs as reported in their SPCs and different ICs.

**Design, Setting, and Participants:**

This cross-sectional study was conducted using data from SPCs for 5 PPIs (omeprazole, esomeprazole, lansoprazole, pantoprazole, and rabeprazole) and 5 ICs (ie, INTERCheck WEB, Micromedex, Lexicomp, Epocrates, and drugs.com). Information from the SPCs and the ICs were extracted between July 15 and 30, 2023.

**Main Outcomes and Measures:**

The main outcome was the level of agreement among SPCs and the 5 ICs in identifying drugs potentially interacting with PPIs and attributing drug-drug interaction (DDI) severity categories. The level of agreement was computed using Gwet AC1 statistic on the 5 ICs and by comparing 4-sets and 2-sets of ICs. As a sensitivity analysis, the level of agreement in listing PPI-related DDIs was evaluated using Cohen κ and Fleiss κ coefficients.

**Results:**

Considering SPCs and the 5 ICs, a total of 518 potentially interacting drugs with omeprazole were reported, 455 for esomeprazole, 433 for lansoprazole, 421 for pantoprazole, and 405 for rabeprazole. As compared with the ICs, the SPCs reported a much smaller number of drugs potentially interacting with PPIs, with proportions ranging from 2.7% (11 potentially interacting drugs) for rabeprazole to 7.6% (33 potentially interacting drugs) for lansoprazole of the total identified drugs at risk of interaction with a PPI. The overall level of agreement among the 5 ICs for identifying potential interactions was poor (from 0.23 [95% CI, 0.21-0.25] for omeprazole to 0.27 [95% CI, 0.24-0.29] for pantoprazole and 0.27 [95% CI, 0.25-0.29] for rabeprazole). Similarly, the level of agreement was low in 4-set and 2-set analyses as well as when restricting the analysis to the potential DDIs identified as severe (range, 0.30-0.32).

**Conclusions and Relevance:**

This cross-sectional study found significant disagreement among different ICs and SPCs, highlighting the need to focus on standardizing DDI databases. Therefore, to ensure evaluation and prevention of clinically relevant DDIs, it is recommended to revise multiple ICs and consult with specialists, such as clinical pharmacologists, particularly for patients with complex medical conditions.

## Key Points

**Question:**

What is the level of agreement between the summary of product characteristics (SPCs) and interaction checkers (ICs) in providing information concerning drug-drug interactions (DDIs) related to proton pump inhibitors?

**Findings:**

In this cross-sectional study of the SPCs for 5 proton pump inhibitors and 5 ICs, significant inconsistencies in listing potential pharmacological interactions between SPCs and ICs, as well as among different ICs, were found. Similarly, the level of agreement was low in 4-set and 2-set analyses as well as when restricting the analysis to the potential DDIs identified as severe.

**Meaning:**

In this study, large heterogeneity of ICs and SPCs in reporting information on potential DDIs with proton pump inhibitors was found, highlighting the need to create a criterion standard DDI dataset.

## Teaser

This cross-sectional study evaluates the consistency between summary of product characteristics and interaction checkers for potential drug-drug interactions with proton pump inhibitors (PPIs).

## Introduction

Proton pump inhibitors (PPIs) are among the most commonly prescribed drug classes and are used for both the prevention of nonsteroidal anti-inflammatory drug (NSAID)–induced ulcers and the treatment of acid-related disorders (eg, gastroesophageal reflux disease and peptic ulcer disease). A large body of evidence documented an increased risk of severe adverse drug reactions (ADRs), especially with long-term and inappropriate use of these drugs, also due to pharmacokinetic and pharmacodynamic drug-drug interactions (DDIs).^1,2,3^

PPIs may alter the absorption and bioavailability of medications with pH-dependent solubility by increasing gastric pH. Furthermore, PPIs are mainly metabolized in the liver by the cytochrome P450 (CYP) enzyme system, particularly CYP2C19 and CYP3A4, thus potentially modifying the metabolism of coadministered drugs via competitive inhibition. However, intraclass differences in CYP affinity exist. In particular, omeprazole has been shown to carry a relevant potential for DDIs due to its higher affinity for CYP2C19 and moderate affinity for CYP3A4, while pantoprazole has the lowest potential for pharmacokinetic interactions. Furthermore, compared with omeprazole, lansoprazole, esomeprazole, and rabeprazole exhibit a weaker potential for DDIs, although they have been less extensively investigated.^3,4^

PPI-related DDIs can lead to serious adverse effects, such as an increased risk of fractures, impaired absorption of nutrients, susceptibility to severe infections, and reduced effectiveness of coadministered medications.^5^ The risk of PPI-related ADRs and DDIs is particularly increased among older patients, who are more likely to be affected by multimorbidity and receive polypharmacy.^6^ As such, to prevent potential ADRs due to DDIs, it is of paramount importance for clinicians to have access to complete and precise information about potentially interacting coprescriptions, especially in older patients receiving many concomitant medications.^7^ This information can be retrieved from the summary of product characteristics (SPCs) as well as drug-drug interaction checkers (ICs), which are databases accessible online for health care professionals. However, the consistency and accuracy of SPCs and different ICs for identifying DDIs can vary significantly.^8,9^ Regarding PPIs specifically, a study^10^ published in 2006 demonstrated that the information on potential DDIs with PPI significantly changed according to the information source. To our knowledge, this is the only study that investigated the level of agreement among different information sources concerning potential DDIs with PPIs. It found poor agreement in identifying potential DDIs between the SPCs and a widely used IC.^10^ Additionally, this study showed that 9% of PPI users were coprescribed PPIs and potentially interacting drugs during the year 2003 in a local health unit of Southern Italy.^10^

The aims of the present study were to assess the level of agreement among 5 different ICs accessible by health care professionals for identifying medicines at interaction risk for omeprazole, esomeprazole, lansoprazole, pantoprazole, and rabeprazole and classifying the severity of PPI-related DDIs. Additionally, we aimed to evaluate the consistency of the information reported by each IC with the PPIs’ SPCs.

## Methods

This cross-sectional study was conducted in accordance with the Strengthening the Reporting of Observational Studies in Epidemiology (STROBE) reporting guideline. Ethical approval and informed consent was not required because this research did not involve human participants or animals.

### Data Sources

#### ICs

In this cross-sectional study, 3 subscription drug ICs (ie, INTERCheck WEB [Istituto di Ricerche Farmacologiche Mario Negri], IC 1^11^; Micromedex [IBM Corp], IC 2^12^; and Lexicomp [Wolters Kluwer], IC 3^13^) and 2 open-access drug ICs (ie, Epocrates [Athenahealth], IC 4^14^; and drugs.com [Cerner Multum], IC 5^15^), commonly utilized by health care professionals, were evaluated. As IC 1 is an Italian IC, the data reported were translated into English to be included in the analysis. These drug ICs were selected based on their utilization worldwide, as documented in previous studies on DDIs.^16,17,18,19^ Additionally, IC 5 is recommended by the US Food and Drug Administration (FDA) as a reliable source for drug information.^20^ Types of information provided and their information sources as stated by IC websites on February 20, 2024, are shown in eTable 1 in Supplement 1.

#### SPCs

SPCs of all PPIs under investigation were retrieved from the official websites of the European Medicines Agency (EMA) and FDA. Each of these SPCs contains a specific section concerning DDIs (paragraph 7 in an FDA SPC and paragraph 4.5 in an EMA SPC), which provides information on potentially interacting drugs and drug classes and DDI-related ADRs. When SPCs reported an entire drug class as being potentially interacting with the specific PPI, all molecules approved by EMA and/or FDA belonging to that class were considered and extracted from the Anatomical Therapeutic Classification (ATC)/DDI index 2024 of the World Health Organization.^21^ When general information about pharmacokinetic or pharmacodynamic characteristics was mentioned (eg, cytochrome inducers or inhibitors), it was not possible to identify specific drugs and therefore no drugs were included in the analysis. As regards SPC information considered in the study, for each PPI, data included in the specific sections of the FDA and EMA SPCs were combined.

### DDI Severity Classification

For each drug under investigation, all 5 ICs provide a comprehensive list of all potentially interacting drugs and detailed information on the mechanisms behind these potential DDIs and their associated severity categories. All ICs classify DDI severity using several comparable categories, but they use different labels. For this reason, the DDI severity categories provided by the different sources were standardized into 4 groups: severe (ie, contraindicated or major DDIs), moderate, minor, and unknown (eTable 2 in Supplement 1). Each IC is generally updated on a weekly basis. In this study, data concerning all drug-PPI interaction pairs were extracted from the 5 ICs in the period between July 15 and July 30, 2023. Concerning SPCs, the same classification used for the ICs could not be applied, since they generally report only descriptive information. As such, the consistency of the information reported in SPCs vs ICs for DDI severity was not evaluated.

### Study Drugs

We included PPIs currently approved for use in Europe by the EMA. There are pantoprazole (ATC, A02BC02), omeprazole (ATC, A02BC01), esomeprazole (ATC, A02BC05), lansoprazole (ATC, A02BC03), and rabeprazole (ATC, A02BC04).

### Statistical Analysis

As a first step, descriptive analyses reported the numbers of drugs identified as potentially interacting with each PPI by each respective SPC and ICs and showed the distribution of potentially interacting drugs across different DDI severity categories assigned by each IC. In particular, the proportion of drugs identified as potentially interacting by each IC was displayed using matrices for each PPI, while the distribution of DDI severity categories was plotted using bar plots.

As primary analysis, the level of agreement among the 5 ICs for the identification of drugs potentially interacting with each PPI (ie, binary variable: interaction or noninteraction) was evaluated using Gwet AC1. The Gwet AC1 statistic represents a chance-corrected measure of interrater reliability, used to evaluate the consensus among multiple raters on categorical and ordinal data. It offers an alternative to the κ statistic, and it is specifically designed to yield more consistent results amidst significant category imbalances (in our context, of severity levels).^22,23^ Additionally, the Gwet AC1 is less affected by the prevalence of the outcomes and exhibits diminished sensitivity to marginal homogeneity, which occurs when raters have different propensities for using certain categories (eg, severe, moderate, minor, or unknown).^24^ The Gwet AC1 coefficient measures observer agreement as follows: 1 represents perfect agreement, 0.76 to 1 signals excellent concordance, 0.41 to 0.75 suggests moderate to good agreement, and 0 to 0.40 indicates poor agreement. Values below 0 denote disagreement, with −1 being complete disagreement. Values near 0 with a nonsignificant *P* value are indicative of an agreement that is not statistically distinguishable from chance.^25^ The same analysis was conducted by comparing groups of 4 ICs (ie, systematically excluding 1 of the 5 ICs from each calculation) and comparing pairs of ICs (ie, systematically excluding 3 of the 5 ICs from each calculation).

Furthermore, the level of agreement among the ICs in attributing DDI severity categories was assessed. This evaluation was also carried out restricting to severe category DDIs for all 5 ICs (ie, including only potential DDIs that were categorized as severe by at least 1 IC under analysis) as well as comparing groups of 4 ICs (ie, systematically excluding 1 of the 5 ICs from each calculation) and comparing pairs of ICs (ie, systematically excluding 3 of the 5 ICs from each calculation).

As sensitivity analysis, we calculated Cohen and Fleiss κ coefficients to assess the agreement in listing DDIs among all 5 ICs, groups of 4, and different pairs. All statistical calculations were computed using the R version 4.2.2 (R Project for Statistical Computing), with packages irrCAC, irr, psych, and fmsb.

## Results

Considering SPCs and 5 ICs, a total of 518 potentially interacting drugs with omeprazole were reported, 455 for esomeprazole, 433 for lansoprazole, 421 for pantoprazole, and 405 for rabeprazole (Table 1). Overall, the IC reporting the highest number of potential DDIs for all PPIs was IC 1, followed by IC 5, IC 3, IC 4, and IC 2 (Table 1). In particular, IC 1 identified more than 55% of the total potentially interacting drugs reported by all ICs for each PPI.

**Table 1.** Potentially Interacting Drugs Identified by PPI SPCs and Each IC, Stratified by PPI and DDI Severity

Interaction	SPC	IC, No. (% of total)										Total, No.
		IC 1		IC 2		IC 3		IC 4		IC 5		
**Pantoprazole**												
Potentially interacting drugs	19 (4.5)	278 (66.0)		51 (12.1)		100 (23.8)		69 (16.4)		144 (34.2)		421
Severe DDI	NA	161 (79.7)		40 (19.8)		32 (15.8)		31 (15.3)		16 (7.9)		202
Moderate DDI	NA	115 (49.6)		10 (4.3)		41 (17.7)		31 (13.4)		109 (47.0)		232
Minor DDI	NA	2 (4.2)		1 (2.1)		27 (56.3)		7 (14.6)		19 (39.6)		48
Drugs reported in both SPC and each IC	14 (73.7)	10 (52.6)		8 (42.1)		11 (57.9)		7 (36.8)		10 (52.6)		19
**Omeprazole**												
Potentially interacting drugs	26 (5.0)	292 (56.4)	89 (17.2)		172 (33.2)		138 (26.6)		201 (38.8)		518	
Severe DDI	NA	172 (72.9)	69 (29.2)		38 (16.1)		39 (16.5)		22 (9.3)		236	
Moderate DDI	NA	118 (42.1)	18 (6.4)		68 (24.3)		66 (23.6)		134 (47.9)		280	
Minor DDI	NA	2 (1.5)	2 (1.5)		66 (50.4)		33 (25.2)		45 (34.4)		131	
Drugs reported in both SPC and each IC	26 (100)	21 (80.8)	20 (76.9)		24 (92.3)		20 (76.9)		20 (76.9)		26	
**Lansoprazole**												
Potentially interacting drugs	33 (7.6)	274 (63.3)	64 (14.8)		111 (25.6)		86 (19.9)		162 (37.4)		433	
Severe DDI	NA	163 (78.7)	44 (21.3)		35 (16.9)		32 (15.5)		20 (9.7)		207	
Moderate DDI	NA	106 (44.2)	17 (7.1)		55 (22.9)		31 (12.9)		118 (49.2)		240	
Minor DDI	NA	5 (7.2)	3 (4.3)		21 (30.4)		23 (33.3)		24 (34.8)		69	
Drugs reported in both SPC and each IC	26 (78.8)	22 (66.7)	14 (42.4)		15 (45.5)		15 (45.5)		18 (54.5)		33	
**Esomeprazole**												
Potentially interacting drugs	27 (5.9)	266 (58.5)	60 (13.2)		115 (25.3)		131 (28.8)		177 (38.9)		455	
Severe DDI	NA	159 (77.6)	44 (21.5)		38 (18.5)		36 (17.6)		21 (10.2)		205	
Moderate DDI	NA	105 (41.3)	15 (5.9)		51 (20.1)		60 (23.6)		123 (48.4)		254	
Minor DDI	NA	2 (2.3)	1 (1.1)		26 (29.9)		35 (40.2)		33 (37.9)		87	
Drugs reported in both SPC and each IC	27 (100)	19 (70.4)	19 (70.4)		23 (85.2)		20 (74.1)		24 (88.9)		27	
**Rabeprazole**												
Potentially interacting drugs	11 (2.7)	267 (65.9)	53 (13.1)		102 (25.2)		67 (16.5)		143 (35.3)		405	
Severe DDI	NA	162 (80.6)	40 (19.9)		32 (15.9)		31 (15.4)		17 (8.5)		201	
Moderate DDI	NA	103 (45.8)	12 (5.3)		42 (18.7)		31 (13.8)		109 (48.4)		225	
Minor DDI	NA	2 (4.3)	1 (2.2)		28 (60.9)		5 (10.9)		17 (37.0)		46	
Drugs reported in both SPC and each IC	11 (100)	10 (90.9)	10 (90.9)		9 (81.8)		8 (72.7)		10 (90.9)		11	

Abbreviations: DDI, drug-drug interaction; IC, interaction checker; NA, not applicable; PPI, proton pump inhibitor; SPC, summary of product characteristic.

**Figure 1.**
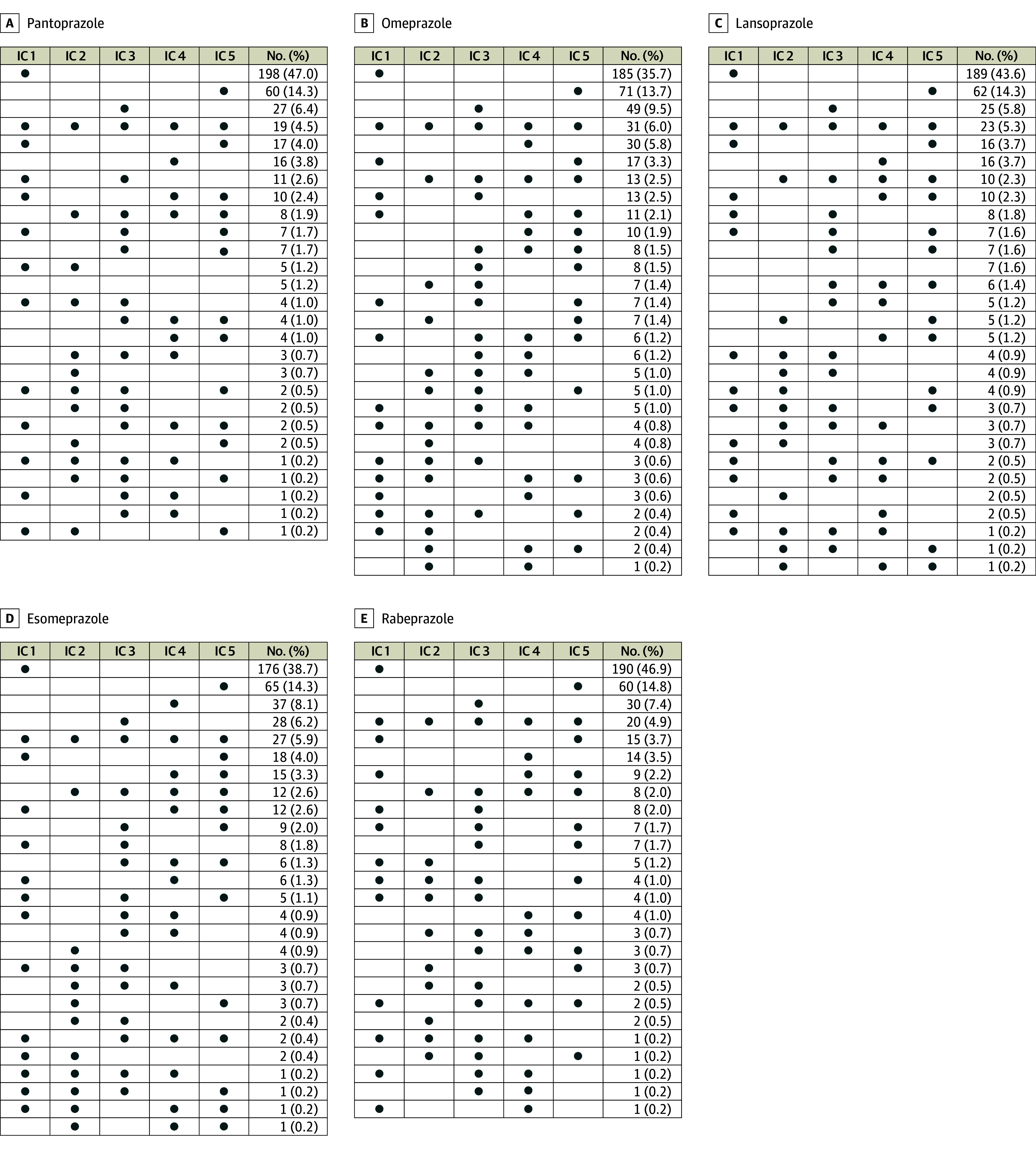
Matrices Showing the Numbers of Drugs Listed as Potentially Interacting With Proton Pump Inhibitors by Each Interaction Checker (IC), Stratified by Proton Pump Inhibitor

Of the total number of drugs reported as interacting with PPIs, only a small proportion was identified simultaneously by all 5 ICs assessed. Specifically, all ICs recognized only 31 medications (6.0% of the total identified) as interacting with omeprazole, 27 (5.9%) with esomeprazole, 23 (5.3%) with lansoprazole, 20 (4.9%) with rabeprazole, and 19 (4.5%) with pantoprazole (Figure 1).

**Figure 2.**
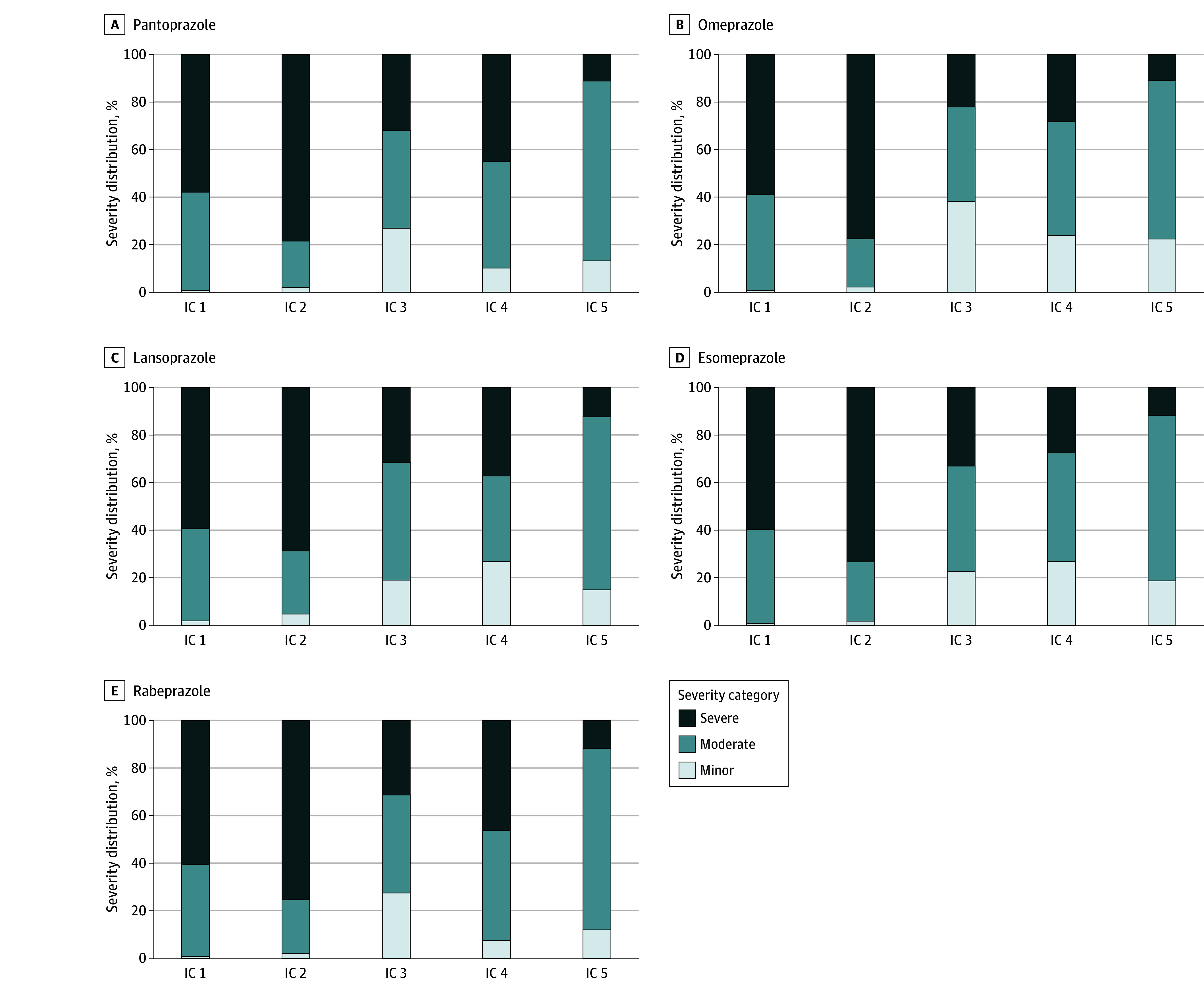
Severity Category Distribution by Interaction Checker (IC) of the 5 Proton Pump Inhibitors Under Study

As compared with the ICs, the SPCs reported a much smaller number of drugs potentially interacting with PPIs, with proportions ranging from 2.7% (11 potentially interacting drugs) for rabeprazole to 7.6% (33 potentially interacting drugs) for lansoprazole of the total identified drugs at risk of interaction with PPI (Table 1). Despite the large number of the identified potentially interacting drugs, each IC captured only a portion of those reported by the SPCs, ranging from 36.8% to 92.3%. In addition, 5 drugs reported as interacting with pantoprazole (ie, acenocoumarol, amprenavir, darunavir, lopinavir, and phenindione) and 7 with rabeprazole (ie, aluminum hydroxide, amprenavir, calcium carbonate, carbamazepine, darunavir, lopinavir, and sodium bicarbonate) by the related SPCs were not identified by any of the ICs (Table 1). Regarding the classification of DDIs’ severity, substantial differences between different ICs were observed. Specifically, the majority of the DDIs reported by IC 2 and IC 1 were classified as severe (range: 68.8%-78.4% and 57.9%-60.7%, respectively), while the severity category more frequently reported by IC 3 and IC 5 was moderate (range: 39.5%-49.5%, respectively) (Table 1 and Figure 2).

The overall level of agreement among the 5 different ICs for identifying potentially interacting drugs was poor, and it ranged from 0.23 (95% CI, 0.21-0.25) for omeprazole to 0.27 (95% CI, 0.24-0.29) for pantoprazole and 0.27 (95% CI, 0.25-0.29) for rabeprazole (Table 2 and Table 3). When comparing different groups of 4 ICs, the analysis yielded lower scores for all PPIs under study, ranging from 0.06 to 0.10 (eTable 3 in Supplement 1). Regarding the pairwise comparison of ICs, all Gwet AC1 scores were lower than 0 (indicating chance agreement or significant disagreement), ranging from −0.77 for the IC 1-IC 4 pair in pantoprazole evaluation to −0.06 for the IC 3-IC 2 pair in both lansoprazole and esomeprazole evaluations (eTable 3 in Supplement 1).

**Table 2.** Illustrative Examples of Listing and Categorizing Potential DDIs Among SPCs and Different Interaction Checkers

Drug name	SPCs	IC 1	IC 2	IC 3	IC 4	IC 5
Pantoprazole						
Amiodarone	Not listed	Severe DDI	Not listed	Not listed	Not listed	Not listed
Captopril	Not listed	Not listed	Not listed	Not listed	Severe DDI	Not listed
Indinavir	Listed	Severe DDI	Not listed	Moderate DDI	Not listed	Moderate DDI
Omeprazole						
Methadone	Not listed	Severe DDI	Not listed	Not listed	Severe DDI	Not listed
Trazodone	Not listed	Severe DDI	Not listed	Not listed	Not listed	Minor DDI
Triazolam	Not listed	Not listed	Moderate DDI	Moderate DDI	Severe DDI	Moderate DDI
Lansoprazole						
Azithromycin	Listed	Severe DDI	Not listed	Not listed	Not listed	Not listed
Fluoxetine	Not listed	Moderate DDI	Not listed	Moderate DDI	Minor DDI	Not listed
Terlipressine	Not listed	Severe DDI	Not listed	Not listed	Not listed	Not listed
Esomeprazole						
Cisplatin	Not listed	Not listed	Not listed	Not listed	Not listed	Moderate DDI
Diazepam	Listed	Not listed	Moderate DDI	Minor DDI	Moderate DDI	Moderate DDI
Digoxin	Listed	Moderate DDI	Moderate DDI	Minor DDI	Moderate DDI	Moderate DDI
Rabeprazole						
Ibandronate	Not listed	Moderate DDI	Not listed	Moderate DDI	Not listed	Not listed
Methotrexate	Listed	Severe DDI	Severe DDI	Severe DDI	Moderate DDI	Severe DDI
Mycophenolate	Listed	Moderate DDI	Severe DDI	Moderate DDI	Minor DDI	Moderate DDI

Abbreviations: DDI, drug-drug interaction; SPC, summary of product characteristic.

**Table 3.** Agreement Level in Listing and Severity Categorization of Potentially Interacting Drugs Across 5 Interaction Checkers

Category	Agreement (95% CI)^a^	*P* value
**Pantoprazole**		
Interacting vs noninteracting	0.27 (0.24-0.29)	<.001
All categories	0.46 (0.44-0.47)	<.001
Restricting to severe category	0.32 (0.30-0.35)	<.001
**Omeprazole**		
Interacting vs noninteracting	0.23 (0.21-0.25)	<.001
All categories	0.42 (0.40-0.43)	<.001
Restricting to severe category	0.30 (0.27-0.33)	<.001
**Lansoprazole**		
Interacting vs noninteracting	0.25 (0.22-0.27)	<.001
All categories	0.43 (0.41-0.45)	<.001
Restricting to severe category	0.31 (0.28-0.34)	<.001
**Esomeprazole**		
Interacting vs noninteracting	0.25 (0.22-0.27)	<.001
All categories	0.44 (0.42-0.46)	<.001
Restricting to severe category	0.30 (0.27-0.34)	<.001
**Rabeprazole**		
Interacting vs noninteracting	0.27 (0.25-0.29)	<.001
All categories	0.46 (0.44-0.48)	<.001
Restricting to severe category	0.32 (0.29-0.35)	<.001

^a^ For Gwet AIC, 1 indicates perfect agreement; 0.76 to 1, excellent agreement; 0.41 to 0.75, intermediate to good agreement; 0 to 0.40, poor agreement; less than 0, disagreement; and −1, complete disagreement. Values around 0 with nonsignificant *P* values indicate agreement no different from chance.

The overall level of agreement among the 5 ICs in categorizing the severity of potential DDIs was moderate, with Gwet AC1 statistic values ranging from 0.42 (95% CI, 0.40-0.43) for omeprazole to 0.46 (95% CI, 0.44-0.47) for pantoprazole and 0.46 (95% CI, 0.44-0.48) for rabeprazole (Table 3). When restricting the analysis to the potential DDIs identified as severe by at least 1 IC, the level of agreement among all ICs was very low, and it was comparable for all 5 PPIs under study (range, 0.30-0.32) (Table 3). The overall level of agreement was lower when comparing different groups of 4 ICs as well as different pairs of ICs (eTable 4 in Supplement 1).

In the sensitivity analysis, Fleiss κ yielded chance agreement (pantoprazole, κ = 0.02; omeprazole, κ = 0.06; lansoprazole, κ = 0.04; esomeprazole, κ = 0.05; and rabeprazole, κ = 0.03) in listing DDIs (eTable 5 in Supplement 1). Chance agreement or significant disagreement was found in all other comparisons.

## Discussion

To our knowledge, this is the first study to assess the level of agreement among 5 different commonly utilized ICs and SPCs in identifying drugs that are potentially interacting with PPIs. When prescribing these drugs, physicians should consider the importance of the clinical consequences of PPI-related DDIs, including, among others, the reduced absorption of drugs that require an acidic environment for optimal absorption (eg, ketoconazole, itraconazole, and some antiviral medications such as atazanavir, darunavir, and lopinavir).^2^

The observed heterogeneity and the inconsistencies in the number of potential DDIs reported by different ICs and the SPCs highlight the challenges that are faced by health care professionals when assessing the interaction risk and related safety of medication regimens, especially in populations at higher risk, such as elderly with multimorbidity receiving polypharmacy. Notably, the primary analysis showed overall a poor agreement among the 5 ICs in identifying drugs potentially interacting with PPIs, with Gwet AC1 scores indicating low interrater reliability (range, 0.23-0.27). As an example, methadone was identified as severely interacting with omeprazole by IC 1 and IC 4, whereas other ICs did not list this interaction. These findings are in line with previously published studies examining other ICs, drug classes, or patient populations.^8,10,26,27,28,29^ A study by Trifirò et al^10^ found a low interrater agreement of 0.23 (95% CI, 0.21-0.25) between SPC and IC 2 in evaluating potential DDIs for PPIs.^10^ Similarly, another study assessing the concordance of 6 ICs (ie, ICs 2-5, Clinical Pharmacology Drug Interaction, and Medscape)^30^ in detecting potential DDIs for psychiatric drugs found a poor overall level of agreement of 0.27.

In our study, the low level of agreement in detecting potential DDIs was also observed in the categorization of potentially severe DDIs (range, 0.30-0.32), which is the most clinically relevant information. For instance, mycophenolate was identified as interacting with rabeprazole by all ICs. However, while IC 1, IC 3, and IC 5 categorized such interaction as moderate, IC 4 classified it as minor and IC 2 as severe. Similarly, a study evaluating the level of agreement in classifying major DDIs among 4 interaction tools, namely Drug Interaction Facts, Drug Interactions: Analysis and Management, Evaluations of Drug Interactions, and IC 2, found a chance agreement of −0.09.^31^ Such findings raise concerns, as it implies that the choice of the IC can significantly affect the identification of potential DDIs, with considerable implications in preventing ADRs and ensuring patient safety.

The heterogeneity among the ICs included in the study may be attributed to several factors, including differences in the algorithms used to detect interactions, the sources of information they draw upon, and the frequency with which the databases are updated.^32^ In particular, IC 1 consistently reported the highest number of potential DDIs, thus potentially reflecting a more inclusive database or a more sensitive interaction detection algorithm. Nevertheless, an increased frequency of identified potential DDIs does not necessarily imply a greater reliability or clinical relevance in supporting clinicians in the decision process at the time of prescription. It may lead to a higher number of marginally relevant or false-positive clinical alerts, posing a risk of alert fatigue among clinicians.^33^ Indeed, presenting clinicians with an excessively long list of potential DDIs, many of which may be of minor significance, can lead to information overload. This, in turn, could dilute attention from more critical interactions, thereby complicating decision-making processes rather than facilitating them. It has been demonstrated that the proportions of alerts of potential DDIs that are ignored or overridden range between 49% and 96%, thus resulting in potential patient harm.^34,35^ On the contrary, it should also be considered that choosing not to prescribe a drug erroneously identified as interacting might deprive patients of the benefit of that medicine.

The disagreement among the ICs raises questions about the criteria used to classify DDIs and highlights the need for standardized methods to evaluate and report DDIs. It is also worth noting that the SPCs report fewer interacting drugs than the ICs, which could be attributed to a different approach of regulatory documents or a delay in updating these documents compared with the more dynamically evolving online tools. SPCs mainly focus on the clinical relevance of DDIs, often tailored to specific drugs, offering detailed explanations of pharmacokinetics, pharmacodynamics, and their clinical implications.^36^ In contrast, ICs frequently refer to entire pharmacological classes or generically to drugs metabolized by a specific cytochrome, which may not readily support clinicians in preventing ADRs.^29,32,33^

It is also noteworthy that the overall level of agreement was higher when including all 5 ICs in the analysis (range, 0.23 to 0.27) than when the analysis was restricted to groups of 4 ICs (range, 0.06 to 0.10) or to pairwise comparisons (range, −0.77 to −0.06). The level of agreement among higher numbers of ICs might be increased due to an increased probability that they identify the same interactions by chance. Additionally, certain ICs may tend to agree with each other, thus compensating for the disagreement of others. When all 5 ICs were compared, these compensatory agreements might inflate the overall agreement measure.^25,37^

The findings of this study point to a need for improved harmonization between different drug information, especially concerning DDIs. Health care professionals rely on accurate and up-to-date information to make informed decisions on patient care. This is especially important in the context of medication review and deprescribing, in which evaluating DDIs plays a pivotal role in optimizing therapeutic strategies and ensuring patient safety.^38^ As such, the discrepancies identified in this study underscore the importance of critically evaluating information from ICs, considering multiple sources when possible, and consulting specialists, such as clinical pharmacologists, who may support physicians with clinical judgment in the final decision-making process.

Future efforts should focus on standardizing DDI databases, improving the algorithms for DDI detection, and ensuring the timely update of both ICs and SPCs. Collaborative efforts among database providers, regulatory agencies, and health care professionals are essential to enhance the reliability of DDI information, ultimately improving patient care and safety. Additionally, such tools should provide information also concerning the frequency and the clinical relevance of the potential DDIs detected. In this regard, real-world studies using both longitudinal health care databases and spontaneous reporting system databases may significantly contribute to generating such evidence.

### Strengths and Limitations

One of the key strengths of our study is its systematic approach in evaluating the identification and severity of DDIs as documented in SPCs and 5 globally utilized ICs. Moreover, to our knowledge this is the first study comparing ICs using the Gwet AC1 statistic. Notably, the use of Gwet AC1, rather than Fleiss and Cohen κ, yielded more reliable results in the presence of significant imbalances in the number of total drugs identified and in severity categories.^24,25,37^

However, some limitations are worth mentioning. First, we did not assess the supportive information provided by various ICs, such as explanatory notes and relevant scientific articles. This issue partly arises from the varying referencing systems used by these tools, which often fail to consistently provide such information. Some ICs limit supporting information only to specific medications, whereas other ICs only cite literature reviews or case reports. As a consequence, we were not able to evaluate the clinical relevance of PPI-related DDIs. Second, it is noteworthy that ICs evaluate only 1:1 interactions, thereby excluding potential additive or multiplicative effects due to multiple interactions. This is particularly relevant in older patients with greater frailty who are usually receiving polypharmacy. Third, recognizing the absence of DDIs is as crucial as acknowledging their presence and/or severity. However, ICs typically only list potentially interacting drugs, omitting those that do not. Given the high number of drugs not mentioned, utilizing statistical methods to evaluate concordance on the nonexistence of DDIs might erroneously indicate a high level of agreement among the ICs. This would not accurately represent the effectiveness or reliability of ICs in identifying clinically relevant DDIs. Fourth, many other ICs are available and were not included in our study. However, we selected 5 of the most-used ICs worldwide, and they were provided significantly inconsistent evidence. Furthermore, we did not consider interactions involving dietary supplements, which are typically excluded from SPCs or ICs, because their inclusion in the analysis would have introduced a selection bias in performing interrater reliability analysis.

## Conclusions

In this cross-sectional study, significant discrepancies among different ICs and both EMA and FDA SPCs in the identification and severity categorization of DDIs for PPIs were found. Such inconsistencies highlight the need for standardization and update of the tools used to detect DDIs to ensure the safe and effective use of medications. To evaluate and prevent clinically relevant DDIs, it is recommended to explore multiple ICs and SPCs as well as to consult with specialists, such as clinical pharmacologists, particularly for patients with complex medical conditions.

## Notes

Supplement 1. **eTable 1.** Types of Information Provided and Their Information Sources as Stated by the Interaction Checker Websites on February 20, 2024 **eTable 2.** Reclassification of DDI Severity Categories as Defined by the 5 Drug Interaction Checkers **eTable 3.** Level of Agreement in Listing DDIs Among Groups of 4 ICs and Different Pairs of ICs, Using the Gwet AC1 Statistic **eTable 4.** Level of Agreement in Categorizing DDIs as Severe Among Groups of 4 ICs and Different Pairs of ICs, Using the Gwet AC1 Statistic **eTable 5.** Level of Agreement in Listing DDIs Among All 5 ICs, Groups of 4, and Different Pairs, Using Cohen and Fleiss κ Statistics

Supplement 2.
Data Sharing Statement
