# Supplementary material for: Agreement of Different Drug-Drug Interaction Checkers for Proton Pump Inhibitors
Source: JAMA Netw Open. 2024 Jul 9;7(7):e2419851. doi: 10.1001/jamanetworkopen.2024.19851 (PMC11234238; doi:10.1001/jamanetworkopen.2024.19851)
Supplement: Supplement 1. — eTable 1. Types of Information Provided and Their Information Sources as Stated by the Interaction Checker Websites on February 20, 2024 eTable 2. Reclassification of DDI Severity Categories as Defined by the 5 Drug Interaction Checkers eTable 3. Level of Agreement in Listing DDIs Among Groups of 4 ICs and Different Pairs of ICs, Using the Gwet AC1 Statistic eTable 4. Level of Agreement in Categorizing DDIs as Severe Among Groups of 4 ICs and Different Pairs of ICs, Using the Gwet AC1 Statistic eTable 5. Level of Agreement in Listing DDIs Among All 5 ICs, Groups of 4, and Different Pairs, Using Cohen and Fleiss κ Statistics [file jamanetwopen-e2419851-s001.pdf]

Supplemental Online Content

Carollo M, Crisafulli S, Selleri M, Piccoli L, L'Abbate L, Trifirò G. Agreement of different drug-drug interaction checkers for proton pump inhibitors. *JAMA Netw Open.* 2024;7(7):e2419851. doi:10.1001/jamanetworkopen.2024.19851

eTable 1. Types of Information Provided and Their Information Sources as Stated by the Interaction Checker Websites on February 20, 2024

eTable 2. Reclassification of DDI Severity Categories as Defined by the 5 Drug Interaction Checkers

eTable 3. Level of Agreement in Listing DDIs Among Groups of 4 ICs and Different Pairs of ICs, Using the Gwet AC1 Statistic

eTable 4. Level of Agreement in Categorizing DDIs as Severe Among Groups of 4 ICs and Different Pairs of ICs, Using the Gwet AC1 Statistic

eTable 5. Level of Agreement in Listing DDIs Among All 5 ICs, Groups of 4, and Different Pairs, Using Cohen and Fleiss κ Statistics

This supplemental material has been provided by the authors to give readers additional information about their work.

© 2024 Carollo M et al. *JAMA Network Open.*

eTable 1. Types of Information Provided and Their Information Sources as Stated by the Interaction Checker Websites on February 20, 2024

	Lexicomp	Micromedex	INTERCheck WEB	Epocrates	drugs.com
**Displayed information**	References, including peer-reviewed scientific studies and SPCs.	References, including peer-reviewed scientific studies, categorized by documentation quality (Excellent, Good, Fair, Unknown) for each DDI.	References, including peer-reviewed scientific studies, SPCs, and other sources (e.g., https://crediblemeds.org/, an online resource providing information on the safety of drugs, such as drugs associated with an increased risk of QT prolongation) for only selected cases.	No information provided.	References, including peer-reviewed scientific studies and SPCs.
**Information sources as stated by the websites**	"Lexi-Interact Online combines literature and scientific understanding of drug interactions throughout the world with a state-of-the-art electronic platform."	"The level of the documentation (Excellent, Good, Fair, Unknown) supporting the interaction is also shown for each interaction listed. The summary column on the results page displays brief, high-level interaction information. Complete Drug Interactions subscribers are able to view additional interaction information by clicking the drug name to open interactions details. Additional information may include: clinical management, onset, probable mechanism, literature summaries, and references for this interaction."	"Sources:<br>• Scientific literature (through periodic bibliographic searches on PubMed)<br>• SPCs<br>• Regulatory Agencies' briefing notes."	"The Drug Interactions section of epocrates drug topics includes clinically relevant identified and evidence-based theoretical interactions that are both pharmacokinetic and pharmacodynamic in nature. epocrates' medical information editors go far beyond the prescribing information when researching drug interactions, often extrapolating broad statements involving mechanisms, such as cytochrome P450 (CYP), P-glycoprotein (P-gp), and pharmacodynamic activity, and extending them to carefully researched lists that are maintained to track metabolic and clinical effects. Attributes such as inhibition potency, substrate sensitivity, and therapeutic index are all considered when determining clinical significance."	"The information provided by Drugs.com is supplied by several external sources: Cerner Multum, Micromedex (part of the global Thomson Health Care Information group) and Physicians' Desk Reference.<br>• Cerner Multum Information Services - Consumer Information database and Drug Interaction Checker database.<br>• Micromedex - Advanced Consumer Information database, Care Guide and Image Database.<br>• Physicians' Desk Reference - PDR Consumer Drug Information database.<br>• Review of Natural Products for consumer and health professionals and Professional Patient Advice for health professionals.<br>All medical and pharmaceutical information is compiled by pharmacists, physicians and medical journalists, based on information supplied by pharmaceutical companies, the FDA and various other agencies."

Abbreviations: DDI = Drug-drug interaction; SPCs = Summary of Product Characteristics.

© 2024 Carollo M et al. *JAMA Network Open.*

eTable 2. Reclassification of DDI Severity Categories as Defined by the 5 Drug Interaction Checkers

	DDI severity category as indicated by drug interaction checkers				
**Re-classification**	**Lexicomp**	**Micromedex**	**INTERCheck WEB**	**Epocrates**	**drugs.com**
Severe	X. Avoid combination<br><br>D. Consider therapy modification	Contraindicated<br><br>Major	D. Contraindicated or very serious<br><br>C. Major	Contraindicated<br><br>Avoid/Use Alternative	Major
Moderate	C. Monitor therapy	Moderate	B. Moderate	Monitor/Modify Therapy	Moderate
Minor	B. No action needed	Minor	A. Minor	Caution advised	Minor
Unknown	No known interaction	-	-	-	Unknown

Abbreviations: DDI = Drug-drug interaction.

© 2024 Carollo M et al. *JAMA Network Open.*

eTable 3. Level of Agreement in Listing DDIs Among Groups of 4 ICs and Different Pairs of ICs, Using the Gwet AC1 Statistic

	Agreement (95% CI)	*p*-value
**Pantoprazole**		
**Comparison of groups of 4 ICs**		
W/o Lexicomp	0.09 (0.07, 0.11)	*p*<0.001
W/o Micromedex	0.09 (0.06, 0.11)	*p*<0.001
W/o INTERCheck WEB	0.09 (0.05, 0.13)	*p*<0.001
W/o Epocrates	0.08 (0.05, 0.10)	*p*<0.001
W/o drugs.com	0.10 (0.08, 0.12)	*p*<0.001
**Pairwise comparison of ICs**		
Lexicomp - Micromedex	-0.13 (-0.36, 0.09)	*p*=0.25
Lexicomp - INTERCheck WEB	-0.68 (-0.77, -0.59)	*p*<0.001
Lexicomp - Epocrates	-0.28 (-0.49, -0.08)	*p*=0.006
Lexicomp - drugs.com	-0.39 (-0.55, -0.23)	*p*<0.001
Micromedex - INTERCheck WEB	-0.76 (-0.85, -0.68)	*p*<0.001
Micromedex - Epocrates	-0.16 (-0.42, 0.09)	*p*=0.21
Micromedex - drugs.com	-0.53 (-0.69, -0.37)	*p*<0.001
INTERCheck WEB - Epocrates	-0.77 (-0.85, -0.69)	*p*<0.001
INTERCheck WEB - drugs.com	-0.64 (-0.73, -0.55)	*p*<0.001
Epocrates - drugs.com	-0.33 (-0.50, -0.15)	*p*<0.001
**Omeprazole**		
**Comparison of groups of 4 ICs**		
W/o Lexicomp	0.07 (0.05, 0.09)	*p*<0.001
W/o Micromedex	0.07 (0.05, 0.09)	*p*<0.001
W/o INTERCheck WEB	0.08 (0.04, 0.11)	*p*<0.001
W/o Epocrates	0.06 (0.04, 0.08)	*p*<0.001
W/o drugs.com	0.08 (0.06, 0.10)	*p*<0.001
**Pairwise comparison of ICs**		
Lexicomp - Micromedex	-0.12 (-0.29, 0.05)	*p*=0.18
Lexicomp - INTERCheck WEB	-0.59 (-0.68, -0.49)	*p*<0.001
Lexicomp - Epocrates	-0.19 (-0.35, -0.04)	*p*= 0.01
Lexicomp - drugs.com	-0.35 (-0.48, -0.22)	*p*<0.001
Micromedex - INTERCheck WEB	-0.70 (-0.79, -0.61)	*p*<0.001
Micromedex - Epocrates	-0.16 (-0.34, 0.03)	*p*=0.10
Micromedex - drugs.com	-0.34 (-0.49, -0.19)	*p*<0.001
INTERCheck WEB - Epocrates	-0.61 (-0.71, -0.51)	*p*<0.001
INTERCheck WEB - drugs.com	-0.58 (-0.67, -0.48)	*p*<0.001
Epocrates - drugs.com	-0.21 (-0.36, -0.06)	*p*=0.005
**Lansoprazole**		
**Comparison of groups of 4 ICs**		
W/o Lexicomp	0.08 (0.06, 0.10)	*p*<0.001
W/o Micromedex	0.07 (0.05, 0.10)	*p*<0.001
W/o INTERCheck WEB	0.08 (0.03, 0.12)	*p*<0.001
W/o Epocrates	0.07 (0.05, 0.09)	*p*<0.001
W/o drugs.com	0.08 (0.06, 0.11)	*p*<0.001
**Pairwise comparison of ICs**		
Lexicomp - Micromedex	-0.06 (-0.27, 0.15)	*p*=0.57
Lexicomp - INTERCheck WEB	-0.66 (-0.76, -0.57)	*p*<0.001
Lexicomp - Epocrates	-0.14 (-0.33, 0.06)	*p*= 0.17
Lexicomp - drugs.com	-0.35 (-0.50, -0.19)	*p*<0.001

© 2024 Carollo M et al. *JAMA Network Open.*

Micromedex - INTERCheck WEB	-0.72 (-0.81, -0.63)	*p*<0.001
Micromedex - Epocrates	-0.19 (-0.41, 0.04)	*p*=0.10
Micromedex - drugs.com	-0.38 (-0.55,-0.21)	*p*<0.001
INTERCheck WEB - Epocrates	-0.72 (-0.81, -0.64)	*p*<0.001
INTERCheck WEB - drugs.com	-0.60 (-0.70, -0.50)	*p*<0.001
Epocrates - drugs.com	-0.29 (-0.46, -0.12)	*p*<0.001
**Esomeprazole**		
**Comparison of groups of 4 ICs**		
W/o Lexicomp	0.08 (0.05, 0.10)	*p*<0.001
W/o Micromedex	0.07 (0.05, 0.09)	*p*<0.001
W/o INTERCheck WEB	0.09 (0.05, 0.13)	*p*<0.001
W/o Epocrates	0.08 (0.05, 0.10)	*p*<0.001
W/o drugs.com	0.10 (0.08, 0.12)	*p*<0.001
**Pairwise comparison of ICs**		
Lexicomp - Micromedex	-0.06 (-0.27, 0.15)	*p*=0.57
Lexicomp - INTERCheck WEB	-0.65 (-0.75, -0.56)	*p*<0.001
Lexicomp - Epocrates	-0.25 (-0.42, -0.08)	*p*=0.005
Lexicomp - drugs.com	-0.36 (-0.51, -0.22)	*p*<0.001
Micromedex - INTERCheck WEB	-0.73 (-0.82, -0.65)	*p*<0.001
Micromedex - Epocrates	-0.26 (-0.46, -0.07)	*p*=0.008
Micromedex - drugs.com	-0.45 (-0.61, -0.30)	*p*=0.008
INTERCheck WEB - Epocrates	-0.65 (-0.75, -0.56)	*p*=0.008
INTERCheck WEB - drugs.com	-0.60 (-0.70, -0.51)	*p*=0.008
Epocrates - drugs.com	-0.21 (-0.37, -0.06)	*p*=0.006
**Rabeprazole**		
**Comparison of groups of 4 ICs**		
W/o Lexicomp	0.09 (0.07, 0.11)	*p*<0.001
W/o Micromedex	0.09 (0.07, 0.11)	*p*<0.001
W/o INTERCheck WEB	0.09 (0.05, 0.13)	*p*<0.001
W/o Epocrates	0.08 (0.06, 0.11)	*p*<0.001
W/o drugs.com	0.10 (0.08, 0.12)	*p*<0.001
**Pairwise comparison of ICs**		
Lexicomp - Micromedex	-0.07 (-0.30, 0.15)	*p*=0.51
Lexicomp - INTERCheck WEB	-0.67 (-0.77, -0.58)	*p*<0.001
Lexicomp - Epocrates	-0.28 (-0.49, -0.08)	*p*=0.006
Lexicomp - drugs.com	-0.36 (-0.52, -0.20)	*p*<0.001
Micromedex - INTERCheck WEB	-0.74 (-0.83, -0.65)	*p*<0.001
Micromedex - Epocrates	-0.12 (-0.38, 0.13)	*p*=0.34
Micromedex - drugs.com	-0.48 (-0.64, -0.31)	*p*<0.001
INTERCheck WEB - Epocrates	-0.75 (-0.84, -0.67)	*p*<0.001
INTERCheck WEB - drugs.com	-0.63 (-0.73, -0.54)	*p*<0.001
Epocrates - drugs.com	-0.33 (-0.51, -0.16)	*p*<0.001

Abbreviations: CI = Confidence Interval, ICs = Interaction checkers; DDIs = Drug-drug interactions; PPIs = Proton Pump Inhibitors; W/o = Without.

Legend for Gwet's AC1 coefficient interpretation:

- +1: Perfect Agreement
- 0.76 to 1: Excellent Agreement
- 0.41 to 0.75: Intermediate to Good Agreement
- 0 to 0.40: Poor Agreement
- Less than 0: Disagreement
- -1: Complete Disagreement

Note: Values around zero with a non-significant *p*-value indicate agreement no different from chance.

© 2024 Carollo M et al. *JAMA Network Open.*

eTable 4. Level of Agreement in Categorizing DDIs as Severe Among Groups of 4 ICs and Different Pairs of ICs, Using the Gwet AC1 Statistic

	Agreement (95% CI)	*p*-value
**Pantoprazole**		
**Comparison of groups of 4 ICs**		
W/o Lexicomp	0.13 (0.11, 0.16)	*p*<0.001
W/o Micromedex	0.14 (0.12, 0. 17)	*p*<0.001
W/o INTERCheck WEB	0.19 (0.06, 0.32)	*p*=0.006
W/o Epocrates	0.13 (0.11, 0.16)	*p*<0.001
W/o drugs.com	0.12 (0.10, 0.15)	*p*<0.001
**Pairwise comparison of ICs**		
Lexicomp - Micromedex	0.20 (-0.13, 0.53)	*p*=0.23
Lexicomp - INTERCheck WEB	-0.89 (-1.00, -0.81)	*p*<0.001
Lexicomp - Epocrates	0.20 (-0.15, 0.55)	*p*=0.26
Lexicomp - drugs.com	0.20 (-0.21, 0.61)	*p*=0.33
Micromedex - INTERCheck WEB	-0.81 (-0.91, -0.72)	*p*<0.001
Micromedex - Epocrates	-0.12 (-0.46, 0.22)	*p*=0.48
Micromedex - drugs.com	-0.03 (-0.42, 0.35)	*p*=0.86
INTERCheck WEB - Epocrates	-0.91 (-0.97, -0.85)	*p*<0.001
INTERCheck WEB - drugs.com	-0.90 (-0.97, -0.83)	*p*<0.001
Epocrates - drugs.com	-0.08 (-0.50, 0.35)	*p*=0.71
**Omeprazole**		
**Comparison of groups of 4 ICs**		
W/o Lexicomp	0.11 (0.09, 0.14)	*p*<0.001
W/o Micromedex	0.12 (0.09, 0.15)	*p*<0.001
W/o INTERCheck WEB	0.12 (0.04, 0.20)	*p*=0.002
W/o Epocrates	0.11 (0.09, 0.14)	*p*<0.001
W/o drugs.com	0.10 (0.07, 0.13)	*p*<0.001
**Pairwise comparison of ICs**		
Lexicomp - Micromedex	-0.02 (-0.29, 0.26)	*p*=0.91
Lexicomp - INTERCheck WEB	-0.86 (-0.94, -0.78)	*p*<0.001
Lexicomp - Epocrates	0.09 (-0.23, 0.42)	*p*=0.57
Lexicomp - drugs.com	0.12 (-0.25, 0.49)	*p*=0.53
Micromedex - INTERCheck WEB	-0.78 (-0.88, -0.60)	*p*<0.001
Micromedex - Epocrates	-0.36 (-0.61, -0.11)	*p*=0.005
Micromedex - drugs.com	-0.24 (-0.52, 0.05)	*p*=0.10
INTERCheck WEB - Epocrates	-0.88 (-0.95, -0.81)	*p*<0.001
INTERCheck WEB - drugs.com	-0.86 (-0.94, -0.78)	*p*<0.001
Epocrates - drugs.com	-0.22 (-0.57, 0.14)	*p*=0.22
**Lansoprazole**		
**Comparison of groups of 4 ICs**		
W/o Lexicomp	0.12 (0.10, 0.15)	*p*<0.001
W/o Micromedex	0.13 (0.10, 0.15)	*p*<0.001
W/o INTERCheck WEB	0.12 (0.01, 0.23)	*p*=0.03
W/o Epocrates	0.12 (0.09, 0.15)	*p*<0.001
W/o drugs.com	0.11 (0.08, 0.14)	*p*<0.001
**Pairwise comparison of ICs**		
Lexicomp - Micromedex	0.12 (-0.20, 0.43)	*p*=0.47
Lexicomp - INTERCheck WEB	-0.86 (-0.94, -0.78)	*p*<0.001
Lexicomp - Epocrates	0.17 (-0.17, 0.52)	*p*=0.31
Lexicomp - drugs.com	-0.01 (-0.40, 0.38)	*p*=0.96

© 2024 Carollo M et al. *JAMA Network Open.*

Micromedex - INTERCheck WEB	-0.78 (-0.88, -0.68)	*p*<0.001
Micromedex - Epocrates	-0.26 (-0.57, 0.05)	*p*=0.10
Micromedex - drugs.com	-0.06 (-0.42, 0.30)	*p*=0.76
INTERCheck WEB - Epocrates	-0.91 (-0.97, -0.85)	*p*<0.001
INTERCheck WEB - drugs.com	-0.85 (-0.94, -0.76)	*p*<0.001
Epocrates - drugs.com	-0.28 (-0.66, 0.09)	*p*=0.14
**Esomeprazole**		
**Comparison of groups of 4 ICs**		
W/o Lexicomp	0.12 (0.09, 0.15)	*p*<0.001
W/o Micromedex	0.12 (0.10, 0.15)	*p*<0.001
W/o INTERCheck WEB	0.17 (0.05, 0.29)	*p*=0.007
W/o Epocrates	0.12 (0.09, 0.15)	*p*<0.001
W/o drugs.com	0.11 (0.08, 0.14)	*p*<0.001
**Pairwise comparison of ICs**		
Lexicomp - Micromedex	0.24 (-0.06, 0.54)	*p*=0.12
Lexicomp - INTERCheck WEB	-0.85 (-0.93, -0.76)	*p*<0.001
Lexicomp - Epocrates	0.15 (-0.17, 0.48)	*p*=0.35
Lexicomp - drugs.com	0.14 (-0.23, 0.52)	*p*=0.44
Micromedex - INTERCheck WEB	-0.80 (-0.90, -0.71)	*p*<0.001
Micromedex - Epocrates	-0.14 (-0.46, 0.17)	*p*=0.37
Micromedex - drugs.com	0.07 (-0.28, 0.43)	*p*=0.69
INTERCheck WEB - Epocrates	-0.89 (-0.96, -0.81)	*p*<0.001
INTERCheck WEB - drugs.com	-0.86 (-0.95, -0.78)	*p*<0.001
Epocrates - drugs.com	-0.22 (-0.59, 0.15)	*p*=0.24
**Rabeprazole**		
**Comparison of groups of 4 ICs**		
W/o Lexicomp	0.13 (0.11, 0.16)	*p*<0.001
W/o Micromedex	0.14 (0.12, 0.16)	*p*<0.001
W/o INTERCheck WEB	0.19 (0.06, 0.33)	*p*=0.007
W/o Epocrates	0.13 (0.10, 0.15)	*p*<0.001
W/o drugs.com	0.12 (0.09, 0.15)	*p*<0.001
**Pairwise comparison of ICs**		
Lexicomp - Micromedex	0.20 (-0.13, 0.53)	*p*=0.23
Lexicomp - INTERCheck WEB	-0.89 (-0.96, -0.81)	*p*<0.001
Lexicomp - Epocrates	0.20 (-0.15, 0.55)	*p*=0.26
Lexicomp - drugs.com	0.17 (-0.24, 0.58)	*p*=0.42
Micromedex - INTERCheck WEB	-0.80 (-0.90, -0.70)	*p*<0.001
Micromedex - Epocrates	-0.05 (-0.39, 0.29)	*p*=0.75
Micromedex - drugs.com	0.03 (-0.36, 0.41)	*p*=0.90
INTERCheck WEB - Epocrates	-0.90 (-0.97, -0.83)	*p*<0.001
INTERCheck WEB - drugs.com	-0.89 (-0.96, -0.81)	*p*<0.001
Epocrates - drugs.com	-0.01 (-0.43, 0.42)	*p*=0.98

Abbreviations: CI = Confidence Interval; DDIs = Drug-Drug Interactions; ICs = Interaction Checkers; PPIs = Proton Pump Inhibitors; W/o = Without.

Legend for Gwet's AC1 coefficient interpretation:

- +1: Perfect Agreement
- 0.76 to 1: Excellent Agreement
- 0.41 to 0.75: Intermediate to Good Agreement
- 0 to 0.40: Poor Agreement
- Less than 0: Disagreement
- -1: Complete Disagreement

Note: Values around zero with a non-significant *p*-value indicate agreement no different from chance.

© 2024 Carollo M et al. *JAMA Network Open.*

eTable 5. Level of Agreement in Listing DDIs Among All 5 ICs, Groups of 4, and Different Pairs, Using Cohen and Fleiss κ Statistics

	Agreement (95% CI)	*p*-value
**Pantoprazole**		
**Comparison of all five ICs**	0.02 (-0.04, 0.07)	*p*=0.55
**Comparison of groups of 4 ICs**		
W/o Lexicomp	-0.09 (-0.14, -0.04)	*p*<0.001
W/o Micromedex	-0.08 (-0.12, -0.03)	*p*=0.002
W/o INTERCheck WEB	0.04 (-0.03, 0.11)	*p*=0.28
W/o Epocrates	-0.09 (-0.13, -0.04)	*p*<0.001
W/o drugs.com	-0.08 (-0.14, -0.03)	*p*=0.003
**Pairwise comparison of ICs**		
Lexicomp - Micromedex	-0.20 (-0.31, -0.09)	*p*<0.001
Lexicomp - INTERCheck WEB	-0.35 (-0.44, -0.26)	*p*<0.001
Lexicomp - Epocrates	-0.45 (-0.57, -0.33)	*p*<0.001
Lexicomp - drugs.com	-0.51 (-0.61, -0.40)	*p*<0.001
Micromedex - INTERCheck WEB	-0.13 (-0.20, -0.07)	*p*<0.001
Micromedex - Epocrates	-0.42 (-0.55, -0.28)	*p*<0.001
Micromedex - drugs.com	-0.24 (-0.34, -0.13)	*p*<0.001
INTERCheck WEB - Epocrates	-0.25 (-0.33, -0.17)	*p*<0.001
INTERCheck WEB - drugs.com	-0.51 (-0.61, -0.42)	*p*<0.001
Epocrates - drugs.com	-0.28 (-0.38, -0.17)	*p*<0.001
**Omeprazole**		
**Comparison of all five ICs**	0.06 (0.02, 0.11)	*p*=0.003
**Comparison of groups of 4 ICs**		
W/o Lexicomp	-0.04 (-0.08, 0.01)	*p*=0.12
W/o Micromedex	-0.02 (-0.07, 0.02)	*p*=0.25
W/o INTERCheck WEB	0.06 (0.01, 0.11)	*p*=0.02
W/o Epocrates	-0.04 (-0.09, 0.00)	*p*=0.04
W/o drugs.com	-0.02 (-0.07, 0.02)	*p*=0.33
**Pairwise comparison of ICs**		
Lexicomp - Micromedex	-0.20 (-0.29, -0.12)	*p*<0.001
Lexicomp - INTERCheck WEB	-0.55 (-0.63, -0.46)	*p*<0.001
Lexicomp - Epocrates	-0.46 (-0.54, -0.38)	*p*<0.001
Lexicomp - drugs.com	-0.55 (-0.62, -0.49)	*p*<0.001
Micromedex - INTERCheck WEB	-0.29 (-0.37, -0.20)	*p*<0.001
Micromedex - Epocrates	-0.35 (-0.45, -0.25)	*p*<0.001
Micromedex - drugs.com	-0.24 (-0.33, -0.15)	*p*<0.001
INTERCheck WEB - Epocrates	-0.44 (-0.53, -0.35)	*p*<0.001
INTERCheck WEB - drugs.com	-0.61 (-0.68, -0.53)	*p*<0.001
Epocrates - drugs.com	-0.41 (-0.49, -0.32)	*p*<0.001
**Lansoprazole**		
**Comparison of all five ICs**	0.04 (0.03, 0.11)	*p*<0.001
**Comparison of groups of 4 ICs**		
W/o Lexicomp	-0.06 (-0.11, -0.02)	*p*=0.008
W/o Micromedex	-0.05 (-0.10, -0.01)	*p*=0.02
W/o INTERCheck WEB	0.05 (-0.01, 0.12)	*p*=0.09
W/o Epocrates	-0.06 (-0.11, -0.01)	*p*=0.02
W/o drugs.com	-0.05 (-0.11, 0.00)	*p*=0.04
**Pairwise comparison of ICs**		

© 2024 Carollo M et al. *JAMA Network Open.*

Lexicomp - Micromedex	-0.24 (-0.35, -0.13)	*p*<0.001
Lexicomp - INTERCheck WEB	-0.40 (-0.50, -0.31)	*p*<0.001
Lexicomp - Epocrates	-0.42 (-0.52, -0.32)	*p*<0.001
Lexicomp - drugs.com	-0.48 (-0.57, -0.38)	*p*<0.001
Micromedex - INTERCheck WEB	-0.19 (-0.26, -0.11)	*p*<0.001
Micromedex - Epocrates	-0.43 (-0.55, -0.31)	*p*<0.001
Micromedex - drugs.com	-0.20 (-0.29, -0.11)	*p*<0.001
INTERCheck WEB - Epocrates	-0.32 (-0.41, -0.23)	*p*<0.001
INTERCheck WEB - drugs.com	-0.56 (-0.64, -0.47)	*p*<0.001
Epocrates - drugs.com	-0.31 (-0.42, -0.21)	*p*<0.001
**Esomeprazole**		
**Comparison of all five ICs**	0.05 (0.00, 0.10)	*p*=0.05
**Comparison of groups of 4 ICs**		
W/o Lexicomp	-0.06 (-0.10, -0.01)	*p*=0.02
W/o Micromedex	-0.04 (-0.09, 0.00)	*p*=0.05
W/o INTERCheck WEB	0.05 (-0.01, 0.12)	*p*=0.08
W/o Epocrates	-0.06 (-0.11, -0.01)	*p*=0.02
W/o drugs.com	-0.04 (-0.09, 0.01)	*p*=0.12
**Pairwise comparison of ICs**		
Lexicomp - Micromedex	-0.18 (-0.27, -0.08)	*p*<0.001
Lexicomp - INTERCheck WEB	-0.43 (-0.52, -0.33)	*p*<0.001
Lexicomp - Epocrates	-0.51 (-0.59, -0.43)	*p*<0.001
Lexicomp - drugs.com	-0.46 (-0.56, -0.36)	*p*<0.001
Micromedex - INTERCheck WEB	-0.18 (-0.26, -0.11)	*p*<0.001
Micromedex - Epocrates	-0.21 (-0.31, -0.11)	*p*<0.001
Micromedex - drugs.com	-0.16 (-0.25, -0.08)	*p*<0.001
INTERCheck WEB - Epocrates	-0.50 (-0.59, -0.40)	*p*<0.001
INTERCheck WEB - drugs.com	-0.61 (-0.69, -0.53)	*p*<0.001
Epocrates - drugs.com	-0.44 (-0.53, -0.36)	*p*<0.001
**Rabeprazole**		
**Comparison of all five ICs**	0.03 (-0.02, 0.08)	*p*=0.27
**Comparison of groups of 4 ICs**		
W/o Lexicomp	-0.08 (-0.13, -0.03)	*p*=0.002
W/o Micromedex	-0.07 (-0.12, -0.02)	*p*=0.007
W/o INTERCheck WEB	0.05 (-0.02, 0.12)	*p*=0.16
W/o Epocrates	-0.07 (-0.12, -0.02)	*p*=0.008
W/o drugs.com	-0.07 (-0.12, -0.01)	*p*=0.02
**Pairwise comparison of ICs**		
Lexicomp - Micromedex	-0.18 (-0.29, -0.07)	*p*<0.001
Lexicomp - INTERCheck WEB	-0.38 (-0.47, -0.28)	*p*<0.001
Lexicomp - Epocrates	-0.42 (-0.55, -0.30)	*p*<0.001
Lexicomp - drugs.com	-0.50 (-0.60, -0.40)	*p*<0.001
Micromedex - INTERCheck WEB	-0.14 (-0.20, -0.08)	*p*<0.001
Micromedex - Epocrates	-0.43 (-0.55, -0.30)	*p*<0.001
Micromedex - drugs.com	-0.22 (-0.33, -0.12)	*p*<0.001
INTERCheck WEB - Epocrates	-0.24 (-0.32, -0.16)	*p*<0.001
INTERCheck WEB - drugs.com	-0.53 (-0.62, -0.44)	*p*<0.001
Epocrates - drugs.com	-0.27 (-0.37, -0.16)	*p*<0.001

Abbreviations: CI = Confidence Interval; DDIs = Drug-Drug Interactions; ICs = Interaction Checkers; PPIs = Proton Pump Inhibitors; W/o = Without.

© 2024 Carollo M et al. *JAMA Network Open.*
