# Supplementary material for: Agreement of Different Drug-Drug Interaction Checkers for Proton Pump Inhibitors
Source: JAMA Netw Open. 2024 Jul 9;7(7):e2419851. doi: 10.1001/jamanetworkopen.2024.19851 (PMC11234238; doi:10.1001/jamanetworkopen.2024.19851)
Supplement: Supplement 2. — Data Sharing Statement [file jamanetwopen-e2419851-s002.pdf]

Data Sharing Statement

Carollo. Agreement of Different Drug-Drug Interaction Checkers for Proton Pump Inhibitors. *JAMA Netw Open*. Published July 09, 2024. doi:10.1001/jamanetworkopen.2024.19851

Data
Data available: No

Additional Information
Explanation for why data not available: Data will be shared on reasonable request.
